# Metastatic lymph node burden impacts overall survival in submandibular gland cancer

**DOI:** 10.3389/fonc.2023.1229493

**Published:** 2023-11-14

**Authors:** Lei Wang, Weihong Shi

**Affiliations:** ^1^ Department of Stomatology, The Third Affiliated Hospital of Xinxiang Medical University, Xinxiang, China; ^2^ Department of Oral Medicine, School of Stomatology, Xinxiang Medical University, Xinxiang, China

**Keywords:** submandibular gland cancer, overall survival, AJCC stage, lymph node metastasis, number of positive lymph nodes

## Abstract

**Objective:**

To assess the effect of the number of positive lymph nodes (LNs) on the overall survival (OS) of patients with submandibular gland cancer (SmGC).

**Methods:**

Patients who had undergone neck dissection for SmGC were retrospectively enrolled in this study. The effect of the American Joint Committee on Cancer (AJCC) N stage, the number of positive LNs, LN size, LN ratio, and extranodal extension (ENE) on OS and recurrence-free survival (RFS) was evaluated using Cox analysis. Prognostic models were proposed based on the identified significant variable, and their performance was compared using hazard consistency and discrimination.

**Results:**

In total, 129 patients were included in this study. The number of positive LNs rather than LN ratio, LN size, and ENE was associated with OS. A prognostic model based on the number of positive LNs (0 vs. 1–2 vs. 3+) demonstrated a higher likelihood ratio and Harrell’s C index than those according to the 7th/8th edition of the AJCC N stage in predicting OS and RFS.

**Conclusions:**

The effect of LN metastasis on OS and RFS was mainly determined by the number of positive LNs. A validation of this finding is warranted in adenoid cystic carcinomas that were not included in this study.

## Introduction

Salivary gland cancer, which accounts for approximately 3–5% of all head and neck cancers, is a relatively uncommon malignancy (1). Neck stage is an important factor that affects disease progression. It is determined by the 8th American Joint Committee on Cancer (AJCC) classification, and it is formulated based on head and neck squamous cell carcinoma (2). However, the two types of tumors show distinct differences in biological behavior (3), which leads to the question of whether the direct application of this classification in salivary gland cancer is possible.

Current literature has proposed alternative lymph node (LN) evaluation methods in patients with salivary gland cancer. Among these methods, the number of positive LNs and extranodal extension (ENE) have shown the greatest potential (4–8). A four-category N stage based on the number of positive LNs (0 vs. 1–2 vs. 3–21 vs. 22+) was proposed by Aro et al. (4). This system provides excellent survival stratification across all histologic types. Similarly, a three-category N stage based on the number of positive LNs and ENE was introduced by Lee et al. (5). This system was superior to the AJCC N stage, enabling a more precise prognostic stratification. Similar results have also been confirmed in other studies (6–8). However, although the two subgroups have apparent differences in proportions and disease prognosis, the origin of cancer from the submandibular and parotid glands was analyzed as one variable in these studies (9). The presence of an additional lymphatic drainage pathway and positive parotid LN in parotid cancer, but no neck LN metastasis, decreases disease control (10). Thus, the relationship of these two factors to the prognosis of submandibular gland cancers (SmGCs) remains unclear.

Therefore, the current study aimed to assess the prognostic significance of LN metastasis burden and ENE in SmGCs.

## Methods

This study was approved by the Xinxiang Medical University Institutional Research Committee (No. CR2021670), and written consent was obtained from all patients before the initial treatment. The study was conducted according to the tenets of the Declaration of Helsinki.

### Study design

The medical records of patients who underwent surgical treatment for SmGCs between January 2000 and December 2022 were retrospectively reviewed. The inclusion criteria were as follows: the disease was primary; neck dissection had been performed; the number of LNs examined was ≥10; and the follow-up data could be obtained via outpatient follow-ups, WeChat, email, telephone, or letters. The demographic characteristics, pathology, treatment, and follow-up information were also collected.

### Study variables

All histopathologic sections were reassessed by two head and neck pathologists to confirm the diagnosis. The tumor and neck stages were graded according to the 7th/8th edition of the AJCC classification. The histologic grade was classified as low, intermediate, and high based on the 5^th^ edition of the World Health Organization Classification of salivary gland tumors. Perineural invasion (PNI) was considered positive if tumor cells entailed either proper perineural or intraneural invasion. Lymphovascular invasion (LVI) was considered positive if tumor cells were present within a lymphovascular vessel. ENE was considered positive if tumor cells were present outside the capsule of the metastatic LN. LN size was defined as the largest diameter of metastatic LNs.

The primary outcome evaluated in this study was the overall survival (OS). The secondary outcome was the recurrence-free survival (RFS). OS time was calculated from the date of surgery to the date of death or the last follow-up; this was censored at 60 months if the duration was longer than five years. RFS time was calculated from the date of surgery to the date of first recurrence or the last follow-up and was censored at 60 months if the duration was longer than five years.

### Treatment principle

Frozen sections of the submandibular gland tumor were obtained routinely in cases where a malignant neoplasm was suspected. Therapeutic neck dissection was performed in cases with pathological or clinically positive LNs. Prophylactic neck dissection was performed in cases with a T3/4 tumor, surrounding tissue invasion, or other adverse features. The extent of neck dissection included at least ipsilateral levels I–III.

### Statistical analysis

The association between the clinicopathologic factors and OS was initially evaluated using univariate analysis. The factors identified as significant in univariate analysis were then assessed using the Cox model. The hazard ratio (HR) of the number of positive LNs, which was assessed as 0 vs. 1 vs. 2 vs. 3 vs. 4+, was calculated to distinguish the effect of different LN metastasis burdens on OS. Subsequently, the optimal cut-off was determined by using binary recursive partitioning analysis (RPA).

Four Cox regression models were constructed during the second analysis. Hazard consistency and discrimination were used to evaluate the two models. Hazard consistency referred to the homogeneity of patients within the same subgroup with similar outcomes; this was reflected by the likelihood ratio. A value of > 0.5 indicated good hazard consistency. On the other hand, hazard discrimination referred to the difference in outcomes between patients of different subgroups with demonstrably different outcomes. It was reflected by Harrell’s C-concordance index. A higher hazard discrimination value indicated better discrimination.

OS and RFS were analyzed using the Kaplan-Meier method and compared using the log-rank test. All analyses were performed using R 3.4.3 (R Core Tea, Vienna, Austria). A p-value0.05 was considered statistically significant.

## Results

### Baseline data

In total, 129 patients (56 men and 73 women; mean age: 48 ± 18 years) were included in this study. The tumor stage was T1 in 15 patients, T2 in 36 patients, T3 in 54 patients, and T4 in 24 patients. The 8th edition of the AJCC N stage was N0 in 59 patients, N1 in 31 patients, N2 in 26 patients, and N3 in 13 patients. The 7th edition of the AJCC N stage was N0 in 59 patients, N1 in 35, N2 in 24, and N3 in 11 patients. ENE was observed in 15 patients, PNI in 27 patients, and LVI in 24 patients. Positive margins were observed in five patients. The most common histopathologic type observed was mucoepidermoid carcinoma (MEC; n=84), followed by myoepithelial carcinoma (n=20) (**Table 1**; **Supplementary Table 1**). The histologic grade was low in 17 patients, intermediate in 75, and high in 37.

**Table 1:** Histologic type distribution of submandibular gland cancer.

Cancer type	N
High grade (n=37)	
Mucoepidermoid carcinoma	22
Duct carcinoma	10
Adenocarcinoma not otherwise specified	5
Intermediate grade (n=75)	
Mucoepidermoid carcinoma	55
Myoepithelial carcinoma	20
Low grade (n=17)	
Mucoepidermoid carcinoma	7
Acinic cell carcinoma	5
Pleomorphic low-grade adenocarcinoma	3
Basal cell carcinoma	1
Epithelial-myoepithelial carcinoma	1

Beyond levels I–III, level IV was dissected in 77 patients. Among these patients, level V was resected in 18 patients. The median and mean number of examined LNs were 29 (range: 11–46) and 28 ± 10, respectively. Among the patients with metastatic disease, 31 had one positive LN, 20 had two positive LNs, 13 had three positive LNs, and 6 had four or more positive LNs. The mean number of positive LNs was 1.9 ± 1.0.

Adjuvant radiotherapy was performed in 87 patients with a median dose of 56 Gy. Among these patients, 28 patients also received adjuvant chemotherapy. The median follow-up duration was 5.3 (range: 0.2–17) years. Forty patients died during the study period; 29 deaths among these were caused by the disease.

### Univariate analysis

In the univariate analysis, tumor stage, the 7th and 8th neck stages, histologic grade, PNI, positive margin, the ratio of positive to total LNs, and treatment were statistically related to OS (Log-rank test, all p<0.05). In contrast, ENE, nodal yield, and level involvement type had no significant effect on OS (Log-rank test, p=0.107, p=0.692, and p=0.554, respectively) (**Table 2**).

**Table 2:** Univariate analysis of predictors for overall survival in submandibular gland cancers.

Factors	p	HR[95%CI]
Age (<50/≥ 50)	0.328	2.87[0.78-6.39]
Sex (Male/female)	0.113	2.16[0.35-20.53]
Tumor stage		
T1		ref
T2	0.432	1.90[0.64-4.28]
T3	0.024	2.35 [1.28-7.69]
T4	<0.001	3.17[1.45-9.24]
7^th^ Neck stage		
N0		ref
N1	0.327	1.90 [0.87-4.67]
N2	0.011	2.89 [1.45-8.73]
N3	<0.001	4.07[1.90-10.37]
Extranodal extension	0.107	3.11[0.85-18.22]
8^th^ Neck stage		
N0		ref
N1	0.425	1.88 [0.69-6.14]
N2	0.010	2.26 [1.37-9.52]
N3	<0.001	4.67[1.66-11.53]
Number of positive lymph nodes		
0		ref
1	0.043	1.86 [1.36-6.48]
2	0.028	2.13 [1.44-8.19]
3	<0.001	4.38 [1.80-10.67]
4+	<0.001	5.39 [2.11-15.26]
Number of positive lymph nodes		
0		ref
1-2	0.034	2.05 [1.42-7.59]
3+	<0.001	5.00 [1.94-14.32]
Pathologic type (Mucoepidermoid cancer/others)	0.489	2.10[0.50-8.66]
Histologic grade		
Low		ref
Intermediate	0.111	2.90 [0.62-13.27]
High	<0.001	6.29[2.01-17.63]
Perineural invasion	0.016	2.97[1.45-8.17]
Lymphovascular invasion	0.247	3.03[0.75-7.26]
Positive margin	0.037	4.17[1.56-8.99]
Level involvement type (I-III/IV-V)	0.554	2.88[0.64-8.22]
Nodal yield (~29/29+)	0.692	3.16[0.42-18.57]
Treatment*		
S		ref
S+R	0.135	1.89 [0.45-7.62]
S+R+C	0.036	2.18[1.26-7.90]

* S, surgery; R, radiotherapy; C, chemotherapy.

### Prognostic model construction

**Figure 1:**
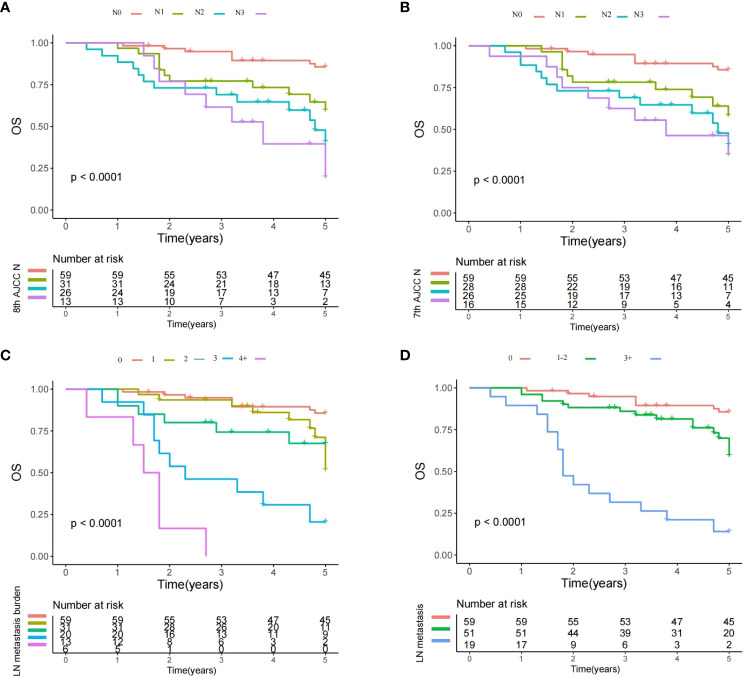
Overall survival plots of different lymph node (LN) status. **(A)** Survival plot for the 8th AJCC N stage: a significant difference existed among the N0, N1, N2, and N3 groups (Log-rank test, p<0.001); **(B)** Survival plot for the 7th AJCC N stage: a significant difference existed among the N0, N1, N2, and N3 groups (Log-rank test, p<0.001); **(C)** Survival plot for the LN metastasis burden: significant difference existed among groups with different metastatic burden (Log-rank test, p<0.001); and **(D)** Survival plot for different number of metastatic LNs: significant difference existed among the different subgroups (Log-rank test, p<0.001).

In multivariate model 1, tumor stage, histologic grade, PNI, positive margin, and treatment were included. The number of positive LNs was associated with OS in the univariate analysis (**Figure 1C**). In the Cox analysis, compared with no LN metastasis, the presence of one and two metastatic LNs showed an HR of 1.89, 95% CI [1.22–3.47] and 2.02 [1.47–5.79], respectively, groups of three and four or more positive LNs had an HR of 4.78 [2.16–10.33] and 5.0 [2.33–18.17], respectively, it is likely that OS decreased with the increase of metastatic LN burden (**Table 3**). Other independent factors included stage T3/4 (2.87 [1.34–5.67], p=0.011; 4.29 [1.91–18.12], p<0.001), high histologic grade (3.18 [1.33–17.58], p<0.001), and positive margin (5.18 [2.02–18.38], p<0.001).

**Table 3:** Prognostic model construction based on different lymph node (LN) evaluation methods.

Multivariate analysis	p	HR [95%CI]
Model 1		
Number of metastatic LNs		
0		ref
1	0.015	1.89 [1.22-3.47]
2	0.003	2.02 [1.47-5.79]
3	<0.001	4.78 [2.16-10.33]
4+	<0.001	5.0 [2.33-18.17]
Tumor stage		
T1		ref
T2	0.244	1.90 [0.76-5.33]
T3	0.011	2.87 [1.34-5.67]
T4	<0.001	4.29 [1.91-18.12]
Histologic grade		
Low		ref
Intermediate	0.117	2.52 [0.54-12.11]
High	<0.001	3.18 [1.33-17.58]
PNI^&^	0.327	2.08 [0.75-8.31]
Positive margin	<0.001	3.18 [1.33-17.58]
Treatment*		
S		ref
S+R	0.522	1.94 [0.57-6.13]
S+R+C	0.275	3.06[0.64-15.43]
Model 2		
Number of metastatic LNs		
0		ref
1-2	0.002	1.99[1.35-4.26]
3+	<0.001	4.98[2.31-16.99]
Tumor stage		
T1		ref
T2	0.278	2.19[0.73-6.16]
T3	0.001	3.91 [1.58-8.43]
T4	<0.001	6.806 [3.12-20.73]
Histologic grade		
Low		ref
Intermediate	0.221	2.08[0.72-12.74]
High	<0.001	5.02 [2.01-18.33]
PNI	0.028	2.12 [1.47-4.87]
Positive margin	<0.001	4.08 [2.13-9.05]
Treatment		
S		ref
S+R	0.517	3.21[0.73-17.22]
S+R+C	0.367	4.05[0.62-20.18]
Model 3		
AJCC 7^th^ N stage		
N0		ref
N1	0.023	1.78 [1.25-4.02]
N2	<0.001	4.21 [1.90-12.64]
N3	<0.001	4.38 [2.05-15.38]
Tumor stage		
T1		ref
T2	0.175	1.99 [0.82-4.57]
T3	0.016	2.33 [1.28-5.44]
T4	<0.001	4.39 [2.12-8.36]
Histologic grade		
Low		ref
Intermediate	0.190	2.04 [0.69-7.43]
High	<0.001	3.22 [1.81-9.13]
PNI	0.011	1.98 [1.22-3.23]
Positive margin	<0.001	5.30 [2.11-16.15]
Treatment		
S		ref
S+R	0.326	2.45 [0.74-6.38]
S+R+C	0.222	3.26 [0.62-9.00]
Model 4		
AJCC 8^th^ N stage		
N0		ref
N1	0.125	1.80[0.825-3.33]
N2	<0.001	6.38 [2.11-17.62]
N3	<0.001	6.77 [2.78-15.37]
Tumor stage		
T1		ref
T2	0.275	1.98 [0.61-5.38]
T3	0.017	2.52 [1.32-6.18]
T4	<0.001	4.30 [2.01-8.75]
Histologic grade		
Low		ref
Intermediate	0.523	3.21 [0.43-8.15]
High	<0.001	4.23 [1.99-10.43]
PNI	0.031	2.12 [1.33-6.44]
Positive margin	<0.001	7.33 [2.67-17.44]
Treatment		
S		ref
S+R	0.633	3.17 [0.73-8.13]
S+R+C	0.524	4.05 [0.62-10.08]

* S, surgery; R, radiotherapy; C, chemotherapy.

& PNI, Perineural invasion.

After RPA analysis, additional subgroups based on the number of metastatic LNs were formulated (model 2; 0 vs. 1–2 vs. 3+). The three subgroups had significantly different OS rates in the univariate analysis (**Figure 1D**). Multivariate model 2 revealed that compared with the no metastasis group, the groups of 1–2 and 3+ positive LNs had a HR of 1.99, 95% CI [1.35–4.26] and 4.98 [2.31–16.99]. The two subgroups also had statistically significant differences in terms of the impact on prognosis indicated by HRs (**Table 3**). Other independent factors included stage T3/4 (3.91 [1.58–8.43], p=0.001/6.806 [3.12–20.73], p<0.001), high histologic grade (5.02 [2.01–18.33], p<0.001), PNI (2.12 [1.47–4.87], p=0.028), and positive margin (4.08 [2.13–9.05], p<0.001). This model demonstrated a likelihood ratio of 0.574 and a Harrell’s C index of 0.703.

### Comparison with the AJCC N stage

Based on the univariate analysis, a multivariate model 3 including tumor stage, neck stage defined by the 7^th^ AJCC neck stage, histologic grade, PNI, positive margin, and treatment was constructed to assess the reliability of the 7th edition of the AJCC N stage in predicting OS. Compared with the N0 stage, LN metastasis significantly decreased the OS. However, the HRs of N2 (4.21 [1.90–12.64]) and N3 (4.38 95% CI [2.05–15.38]) were comparably high (**Figure 1B**; **Table 3**). Other independent factors included stage T3/4 (2.33 [1.28–5.44], p=0.016/4.39 [2.12-8.36], p<0.001), high histologic grade (3.22 [1.81–9.13], p<0.001), PNI (1.98 [1.22–3.23], p=0.011), and positive margin (5.30 [2.11–16.15], p<0.001). This model demonstrated a likelihood ratio of 0.427 and a Harrell’s C index of 0.689.

Another multivariate model 4 was developed to evaluate the reliability of the 8th edition of the AJCC N stage. Compared with the N0 stage, N1 (HR 1.80 95%CI [0.83–3.33]) disease did not significantly alter the OS, and the negative impact of LN metastasis did not occur until the development of N2 (6.38 [2.78–15.37]) disease. The groups of N2 and N3 had analogous HRs (**Figure 1A**; **Table 3**). Other independent factors included stage T3/4 (2.52 [1.32–6.18], p=0.017/4.30 [2.01–8.75], p<0.001), high histologic grade (4.23 [1.99–10.43], p<0.001), PNI (2.12 [1.33–6.44], p=0.031), and positive margin (7.33 [2.67–17.44], p<0.001). This model demonstrated a likelihood ratio of 0.401 and a Harrell’s C index of 0.671.

Both models had inferior likelihood ratios and Harrell’s C indices compared with the model based on the number of metastatic LNs (0 vs. 1–2 vs. 3+).

### Second outcome variable analysis

**Figure 2:**
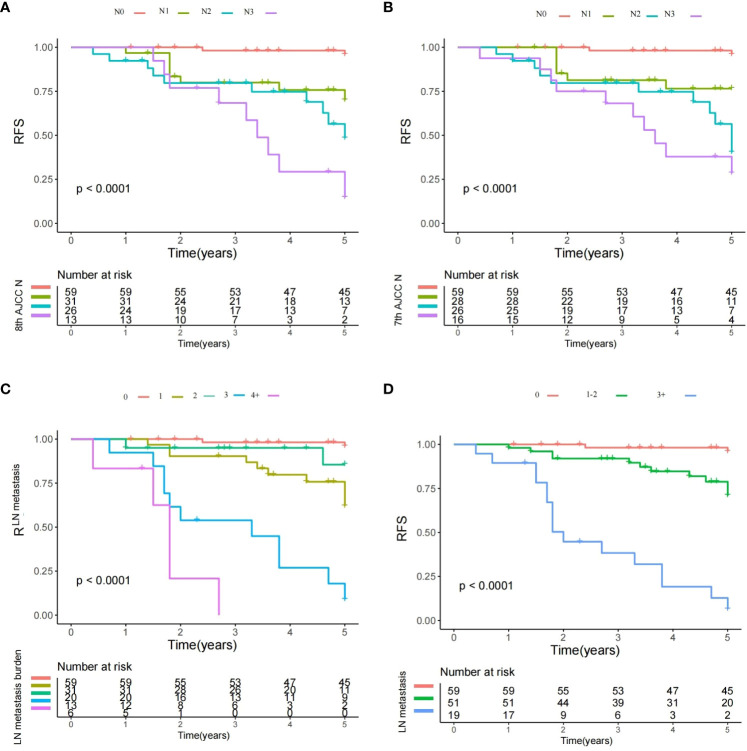
Recurrence-free survival plots of different lymph node (LN) status. **(A)** Survival plot for the 8th AJCC N stage: significant difference existed among the N0, N1, N2, and N3 groups (Log-rank test, p<0.001); **(B)** Survival plot for the 7th AJCC N stage: significant difference existed among the N0, N1, N2, and N3 groups (Log-rank test, p<0.001); **(C)** Survival plot for the LN metastasis burden: significant difference existed among groups with different metastatic burden (Log-rank test, p<0.001); and **(D)** Survival plot for different number of metastatic LNs: significant difference existed among the different subgroups (Log-rank test, p<0.001).

RFS was an essential supplement to OS for prognosis evaluation. All the 7^th^ and 8^th^ AJCC N stages, and LN metastasis burden exhibited a significant impact on RFS (**Figure 2**) (Log-rank test, all with p<0.001). Still, prognostic model based on the number of metastatic LNs (0 vs. 1–2 vs. 3+) showed a likelihood ratio of 0.543 and a Harrell’s C index of 0.689, it was superior to those in models according to the the 7^th^ (likelihood ratio: 0.468; Harrell’s C index: 0.674) and 8^th^ (likelihood ratio: 0.453; Harrell’s C index: 0.645) AJCC N stages.

## Discussion

The most valuable finding in the current study was that the number of metastatic LNs offered better OS stratification than the 7th and 8th editions of the AJCC N stage, it could provide additional information while screening real patients at high risk of mortality.

Neck status is an important prognostic factor as mentioned, and the survival rate could decrease by half even with only one metastatic LN (11, 12). The 7^th^ edition of the AJCC N stage evaluated the number, size, and laterality of positive LNs. In contrast, ENE was considered in the 8^th^ edition of the AJCC N stage (2). Although both stages were formulated based on head and neck squamous cell carcinoma (3), the occurrence of contralateral neck LN metastasis in major salivary gland cancer was very uncommon, and the prognostic significance of ENE has remained controversial (13–15). Therefore, some scholars aimed to develop other alternative N stages. Aro et al. (4) were the first to uncover the phenomenon and enrolled 4520 cases of salivary gland cancers in their study. It was observed that OS worsened without plateauing as the number of metastatic LNs increased. The mortality risk was obvious for those with up to four LNs and then gradually stabilized in those with additional LNs> 4.

Aro et al. might be the first to demonstrate a new LN assessment method based on the number of metastatic LNs (0 vs. 1-2 vs. 3-21 vs. ≥22), its prognostic model exhibited greater accuracy than the 8th edition of the AJCC N stage in predicting OS (4). Lombardi et al. (8) introduced three novel N-classifications according to the number of metastatic nodes (0 vs. 1–3 vs. ≥ 4) and/or their maximum diameter (<20 mm vs. ≥ 20 mm) that showed better performance in OS stratification. Lin et al. (14) showed a three-category LN evaluation method of 1 vs. 2-7 vs. 8+ metastatic LNs exhibited better DSS and OS predictive efficacy than AJCC N stage based on 895 patients with T-4N-3M0 parotid gland carcinoma. Han et al. (15) compared the prognostic value of three models according to the number of metastatic LNs, and found neck classification of 0/1 vs. 2-4 vs. 5+ positive LNs had the best survival prediction in 1689 parotid adenoid cystic cancer patients. Elhusseiny et al. (16) reported that >4 metastatic LNs were associated with worse survival in major salivary gland cancer. Although these studies confirmed the effect of the number of positive LNs on survival in salivary gland cancer, SmGC was not included for analysis (14, 15), or SmGC only accounted for a very small proportion (less than 10%) of this sample size (4, 8, 16). The two main differences, intraglandular LN presence and surgical strategy, between the parotid and submandibular glands, led to the necessity of validating the impact of the number of positive LNs on SmGC.

We noted that the impact was mainly influenced by the number of positive LNs rather than the ratio of positive to total examined LNs or LN size or level involvement type in SmGC. This finding is significant in that it revealed the inadequacy of the AJCC N stage as the presence of one positive LN could indicate N1, N2, or N3 stage in the AJCC N stage; however, patients with one metastatic LN had comparable OS independent of other LN factors. In addition, this study provided the underlying mechanism for explaining the superiority of prognostic model based on the number of metastatic LNs with a higher likelihood ratio and Harrell’s C index.

Nevertheless, conflicted results have been reported by other studies. Cho et al. (17) analyzed the outcome of 99 patients with SmGC. They reported that the ratio of positive to total LNs> 0.15 was related to a nearly 3-fold or higher increase in the risk of locoregional recurrence, distant metastasis, and death. Level IV/V metastasis tended to promote distant metastasis or disease recurrence. However, the authors did not provide the data of the least number of required examined LNs, which prevented further clinical application. Shi et al. (18) divided 376 patients with major salivary gland cancer into three groups: extent 1 referred to level I or parotid LN metastasis, extent 2 referred to level II–IV metastasis, and extent 3 referred to level V or bilateral or rare LN metastasis. Cox analysis revealed clear OS curve separation, whereas the AJCC N classification failed to discriminate the prognosis of the N1 and N2 groups. If the two variables were incorporated into the same Cox analysis, the former would remain an independent prognostic factor, whereas the AJCC N classification would lose significance. We failed to validate the association between the level involvement type and OS, and the difference was partially explained by different inclusion criteria. Unfortunately, no more similar literature was available for comparison.

ENE is another critical prognostic factor that is usually a reliable indicator for the requirement of adjuvant chemotherapy and poor prognosis in patients with head and neck squamous cell carcinoma (19). However, its role in salivary gland cancer has not been studied, and the reported conclusions were contradictory. Lee et al. (5) reported both LN+ number and ENE were independently associated with OS and that the effect of ENE was comparable with that of two or more positive LNs. Their proposed N stage (N0: 0 LN+; N1: 1 LN+; N2: ≥2 LN+ or ENE) had better OS prediction than the 7th/8th edition of the AJCC N staging. However, in a study by Hsieh et al. (13), 51% of the sample developed ENE and had a higher possibility of the incidence of advanced N stage and a greater number of positive LNs, LVI, and PNI. Nevertheless, the OS was like that of those without ENE after adjusting for the number of positive LNs. Comparable results were also described by other authors and us (4, 7, 8, 10, 17), which elucidated that ENE in salivary gland cancer might not demonstrate any influence on survival but was correlated directly with adverse pathologic features that affected the prognosis (10). Thus, the further discussion of the current AJCC N stage and the superiority of our prognostic model based on LN metastasis burden were emphasized.

The current study had some limitations that must be acknowledged. First, there was inherent bias due to the retrospective design of the study. Second, our findings were based on a single constitution; thus, external validation is required before clinical application. Lastly, we did not enroll patients with adenoid cystic carcinoma; hence, it remained unknown whether the finding was suitable for other salivary gland cancers.

In summary, LN metastasis significantly affected OS in SmGC, and the impact was mainly determined by the number of positive LNs rather than other LN factors. A validation of this finding is warranted in adenoid cystic carcinomas that were not included in this study.

## Data availability statement

The original contributions presented in the study are included in the article/**Supplementary Material**. Further inquiries can be directed to the corresponding author.

## Ethics statement

The studies involving humans was approved by Xinxiang Medical University Institutional Research Committee. The studies were conducted in accordance with the local legislation and institutional requirements. The participants provided their written informed consent to participate in this study. Written informed consent was obtained from the individual(s) for the publication of any potentially identifiable images or data included in this article.

## Author contributions

The authors made all the contributions: study design, manuscript writing, selection of studies and study quality evaluation, data analysis, and the revision of the manuscript. All authors contributed to the article and approved the submitted version.
