# Supplementary material for: Metastatic lymph node burden impacts overall survival in submandibular gland cancer
Source: Front Oncol. 2023 Nov 14;13:1229493. doi: 10.3389/fonc.2023.1229493 (PMC10682759; doi:10.3389/fonc.2023.1229493)
Supplement: Supplementary file 1 [file Table_1.doc]

Supplementary Table 1. Detailed information of the enrolled patients.

| Variable | Number |
| --- | --- |
| Age (years) | 48 ± 18 |
| Sex |  |
| Male | 56 |
| Female | 73 |
| Tumor stage |  |
| T1 | 15 |
| T2 | 36 |
| T3 | 54 |
| T4 | 24 |
| 7^th^ Neck stage (N0/N1/N2/N3) |  |
| N0 | 59 |
| N1 | 35 |
| N2 | 24 |
| N3 | 11 |
| Extranodal extension | 15 |
| 8^th^ Neck stage |  |
| N0 | 59 |
| N1 | 31 |
| N2 | 26 |
| N3 | 13 |
| Perineural invasion | 27 |
| Lymphovascular invasion | 24 |
| Positive margin | 5 |
| Level involvement type |  |
| Ⅰ-Ⅲ | 49 |
| Ⅳ-Ⅴ | 11 |
| Number of metastatic lymph nodes |  |
| 0 | 59 |
| 1 | 31 |
| 2 | 20 |
| 3 | 13 |
| 4+ | 6 |
